# TRANVIA: A program for continuum mental health assistance in transition period

**DOI:** 10.1192/j.eurpsy.2023.1526

**Published:** 2023-07-19

**Authors:** L. Pérez Gómez, A. González Álvarez, M. A. Reyes Cortina, E. Lanza Quintana, N. Álvarez Alvargonzález, C. Rodríguez Turiel, E. Lago Machado, J. J. Martínez Jambrina

**Affiliations:** 1 CSM MAGDALENA; 2 Area III, SESPA, AVILES, Spain

## Abstract

**Introduction:**

Transition between adolescence and adulthood represents the most important challenge for personal development and involves several transformations: physical, psychological and social. It is a complex age bracket, concurring the transition from youth psychiatric units to adult ones, with an increased risk for the appearance of mental disorders and risky behaviours. TRANVIA program, developed in Avilés, provides psychiatric assistance to patients between 15 and 25 years old, diagnosed with a severe psychiatric disorder or with an increased risk of having one.

**Objectives:**

Our objectives are: ensuring clinical continuity assistance, promoting communication among professionals and the empowerment of our patients to improve their functionality and quality of life.

**Methods:**

Descriptive study including patients involved in TRANVIA program from November 2019 to November 2021.

**Results:**

During this two-years period there have been 44 referrals to the program, 11 of them were rejected for failure to comply with diagnostic criteria. In November 2021 there were 33 patients included in the TRANVIA program with an average age of 17 years old (range: 15-22). 70% of them were men and 30% women. All of them had psychiatric assistance from different sources: youth mental health units, neuropediatrics… About 75% of the patients were diagnosed with autistic spectrum disorder and approximately three-quarters of the sample needed pharmacological treatment. Risperidone was the most prescribed drug. We have also developed other assistance alternatives as home-based care, relaxation sessions, social worker interventions and coordination with schools.

**Conclusions:**

TRANVIA program has allowed us to provide continual attention to vulnerable patients that shift from youth psychiatric units to adult ones. Patients that meet inclusion criteria were enrolled independently the type of assistance they have previously received. Accessibility and flexibility were our priority. During the described period there was only one dropout, three patients required psychiatric hospitalization and two others visited the emergency department. There have been no cases of completed suicide.

**Disclosure of Interest:**

None Declared

